# Supplementary figures and images for: Acute blood loss in mice forces differentiation of both CD45-positive and CD45-negative erythroid cells and leads to a decreased CCL3 chemokine production by bone marrow erythroid cells
Source: PLoS One. 2024 Sep 4;19(9):e0309455. doi: 10.1371/journal.pone.0309455 (PMC11373861; doi:10.1371/journal.pone.0309455)

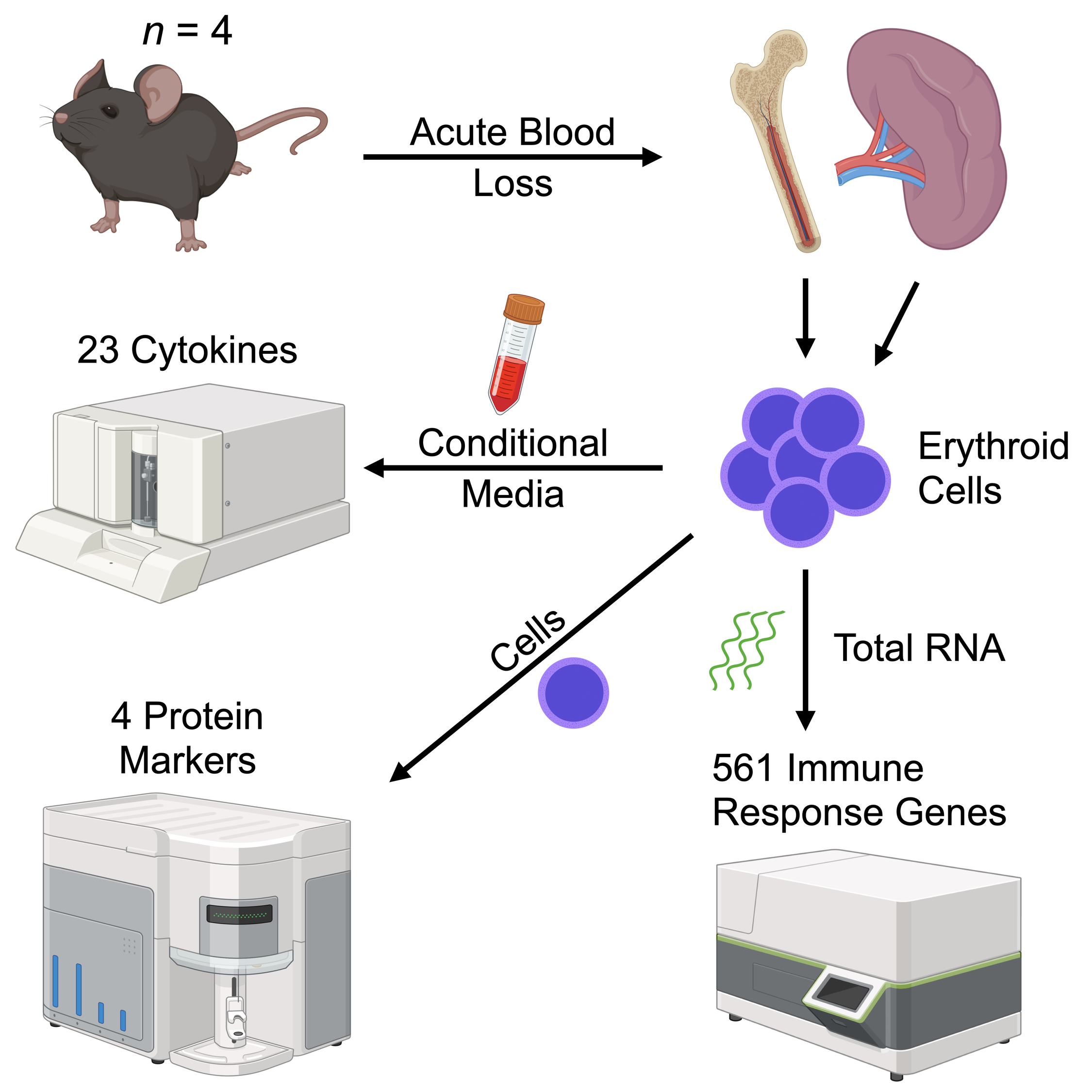

Supplement: S1 Graphical abstract — (TIF) [file pone.0309455.s001.tif]
