# Supplementary material for: Acute blood loss in mice forces differentiation of both CD45-positive and CD45-negative erythroid cells and leads to a decreased CCL3 chemokine production by bone marrow erythroid cells
Source: PLoS One. 2024 Sep 4;19(9):e0309455. doi: 10.1371/journal.pone.0309455 (PMC11373861; doi:10.1371/journal.pone.0309455)
Supplement: Attachment — Submitted filename: Response to Reviewers.docx [file pone.0309455.s002.docx]

Rebuttal letter

Hello

First of all. Thank you for your revision and notes about our manuscript. I wanna respond to your notes.

1. We added some more information about erythropoiesis at acute blood loss conditions. Also, we wanna note that decreasing content of late erythron forms such as orthochomatic erythroblasts and reticulocytes connect with their exit into bloodstream as we suppose. Their exit can be consider like compensatory effect to replace loosed RBC after ABL.
2. Mentioned figures were replaced.
3. List of genes were replaced to the table.
4. We agree that *Clec5a* seems to be just one of the many differentially expressed genes. It need more pointed experiments and functional tests to proof role of this gene in erythron physiology. Now we biased the emphasis to chemokine CCL3, because its mRNA expression and protein production are detected in marrow erythroblasts. We suppose that erythroblasts are potent chemokine-producers and erythroblasts realize immunoregulatory effects via the chemokines.
5. About validation Nanostring (mRNA expression) and Bioplex data (protein production). We agree that in vivo and in vitro results may be different, because in vitro is some idealization model, which facilitate our work and so on. But in vitro model allow see clear affects for example in erythroid cells. We wanna note that we used fresh-isolated erythroid cells for flow cytometry and for Nanostrung analysis. Viability of isolated cells assayed by trypane blue test. Cell cultures were used to next procedures if their viability were 95% or more. At Nanostring analysis we applied more strict criterions to diminish threshold signals and extract these genes which are expressed exactly. And we see that production of protein have accordance with mRNA expression (IL1b, CCL2, CCL3). Also CCL3 expression level is equal Tal1 or CD36 expression level approximately (Tal1 and CD36 are signature genes of erythroid lineage). Although we detected other cytokines and chemokines genes expression we couldn’t check their protein production level by erythroblasts because that molecules (MIF, TGFb1, CXCL12, IL16) didn’t presented in Bioplex panel.

Roman Perik-Zavodskii edited this manuscript in language aspect.
